# Smallholders’ Agricultural Production Efficiency of Conservation Tillage in Jianghan Plain, China—Based on a Three-Stage DEA Model

**DOI:** 10.3390/ijerph17207470

**Published:** 2020-10-14

**Authors:** Xin Yang, Guangyin Shang

**Affiliations:** College of Land Management, Huazhong Agricultural University, Wuhan 430072, China; shanggy@webmail.hzau.edu.cn

**Keywords:** conservation tillage, agricultural production efficiency, three-stage DEA model, smallholders, Jianghan Plain

## Abstract

Based on interviews with 695 smallholders in Jianghan Plain, this paper introduced the three-stage data envelopment analysis (DEA) model to analyze the agricultural production efficiency of conservation tillage adopters and explored the impact of environmental factors on agricultural production efficiency. The empirical results showed the following (1) Planting area, seed consumption, labor input, pesticide usage, chemical fertilizer usage, agricultural film usage were selected as input indicators, agricultural output was chosen as an output indicator, and the traditional DEA model was used to calculate the production efficiency of smallholders, and the agricultural production efficiency of smallholders was found to be at a low level. In addition, environmental and random factors both have significant impacts on efficiency, so they should be stripped. (2) After excluding environmental factors and random factors, the drop in pure technical efficiency of smallholders in the third stage was higher than the drop in scale efficiency when compared with the first stage. Moreover, the true technical efficiency was the main restricting factor for the agricultural production efficiency. (3) Educational level of smallholders, policy support, and information acquisition were the factors that affect the technical efficiency significantly. Improving the efficiency of agricultural production technology for smallholders requires strengthening rural basic education, improving subsidy policies for conservation agricultural technology, and establishing and improving rural information technology services.

## 1. Introduction

Agriculture in China has experienced rapid development during the past several decades. Every year huge amounts of chemical fertilizer and pesticides were put into farmland to guarantee the national grain production and effective supply of agricultural products [1]. This long-lasting highly intensive agricultural production pattern has triggered severe social and ecological problems to the further development of human beings, such as extreme climates [2], environment deterioration [3], biodiversity loss [4] and low agricultural productivity and efficiency [5]. Therefore, it is of vital importance both for the government and growers to discover and implement new agricultural conservation practices to reduce the above consequences resulting from extensive agriculture land uses [1]. Conservation tillage has attracted considerable attention and has been implemented in developed countries such as United States and Australia as it puts equal weight on ecological and economic benefits. Specifically, farmland conservation has been proved to have a promising positive prospect to restrain farmland degradation, reduce greenhouse emission, and improve the quality of agricultural products. Therefore, conservation tillage has been treated as the effective way to facilitate a transformation in agricultural development modes and to achieve the sustainable development of agriculture [6,7,8,9].

Compared with conventional agriculture, the conservation tillage technology spawned to protect the ecology has disadvantages in yield but advantages in price. Under the assumption of “rational man”, the above factors have the dual role of resistance and attraction on the enthusiasm of the smallholders to participate in the farmland protection work [10]. Hence, how to measure the smallholders’ agricultural production efficiency and explore its potential influencing factors is still an unsolved important issue. Therefore, the motivation for this study is to estimate the agricultural production efficiency of smallholders who adopt different levels of conservation tillage and to explore the potential influencing factors of the above agricultural production efficiency. The results of the project will help people to accumulate experience in developing a balance between economy and environment, it can also give a practical answer for whether conservation tillage will have a positive effect on rice production and provide scientific support for the government to design more reasonable policies associated with farmland conservation tillage.

Generally speaking, methods used for measuring production efficiency are classified into two types: parametric methods and non-parametric methods [11]. Due to many advantages, such as not having to preset specific functions and not having to normalize variable units, non-parametric methods have become the mainstream method for evaluating production efficiency in the world [11]. The data envelopment analysis model (DEA) is a non-parametric production efficiency evaluation method that estimates the effective production frontier based on a set of input–output observations [12]. Although DEA is a relatively mature and modern tool to calculate efficiency, it has some shortcomings. The effect of errors on production efficiency cannot be explained by a one-stage DEA model, while external environmental effects cannot be eliminated by the two-stage DEA model [12,13,14]. In order to overcome the above shortcomings, combined with stochastic frontier analysis (SFA) technology, Fried et al. proposed three-stage DEA model [15]. In this model, the agricultural production efficiency of smallholders who adopted conservation is equated with technology adoption, external environmental variables, and random error. The three-stage DEA model has been widely used to calculate the true agricultural production efficiency due to its unique advantages in excluding the impacts from external environmental variables and random error.

Numerous studies conducted on technology adoption have shown that technical efficiency is not only related to the scale of operation, factor allocation, and management level but also affected by technology adoption [16]. Chen et al. pointed out that under the trend of decreasing agricultural planting area in China, applying agricultural technology to productivity plays an important role in improving the efficiency of agricultural production technology and increasing agricultural output [17]. However, will the adoption of agricultural technology affect agricultural production efficiency? In response to this problem, Brummer took Zhejiang as an example and found that the technical efficiency of China’s agricultural production will show an inverted U-shaped characteristic along with technological progress [18]. Hasnah et al. found through research that the excessive adoption of agricultural technology did not increase palm oil production in West Sumatra [19]. On the other hand, Mwalupaso et al. found that adopters of sustainable agricultural technology are more technically efficient than smallholders who have not adopted it [20]. In Nigeria, Alene and Manyong’s research found that the leader of agricultural technology adoption had an advantage in cowpea production compared to the followers of agricultural technology [21]. Technology adoption may increase agricultural production and may also restrict agricultural development. This shows that only an appropriate scale and effective deployment of agricultural technology can improve the technical efficiency of agricultural production. Therefore, the impact of agricultural technology adoption on agricultural production technology efficiency is very necessary. Because, if the adoption of agricultural technology can effectively improve the technical efficiency of agricultural production, then the improvement of agricultural technical efficiency will increase the production efficiency of smallholders and, ultimately, make the scale effect of agricultural production be effective [22].

The above studies have analyzed agricultural technology and technical efficiency in depth, but currently little attention has been paid to agricultural technical efficiency for smallholders who adopt conservation tillage. Moreover, the research methods cannot exclude the influences of environmental factors and random factors, so it is difficult to measure the efficiency accurately. Therefore, taking 695 households in Jianghan Plain as an example, this paper uses the three-stage DEA model to evaluate the agricultural production technical efficiency level of different conservation tillage adopters by eliminating the interference of environmental factors and random factors and explores the impact of environmental variables on the above technical efficiency. It is expected to provide a theoretical reference for local governments to design more smallholder-friendly policies to improve the technology production efficiency and promote the sustainable development of agriculture. The following section describes the data of the variables used. The methods and empirical procedure are presented in the third section. In the fourth section, the results are presented and discussed, and the last section draws conclusions and implications for policy.

## 2. Study Area and Data Collection

### 2.1. Study Area

The study was conducted in Jianghan Plain, which is an important component of the middle and lower reaches of the Yangtze River Plain. With subtropical monsoonal climate, flat terrain, and abundant surface water, the land in Jianghan Plain is extremely fertile. There is 3.02 × 10^5^ hm^2^ of high-quality arable land in Jianghan Plain, which makes it one of the most fundamental basements for rice production in central China. However, the severe social and ecological problems caused by traditional intensive agricultural tillage modes have attracted attention from governments in the Jianghan Plain. Conservation tillage is widely implemented in Jianghan Plain as it offers tremendous environmental benefits by reducing energy consumption and curbing farmland degradation. Five different kinds of conservation agriculture (CA) technologies (straw returning to field, shrimp/fish culture, the different edaphic and climatic characteristics of no-till and less-tillage on farmland, pesticides control techniques, and deep tillage) have been adapted to the edaphic and climatic characteristics of the Jianghan Plain.

Famous for the “shrimp and rice farming” mode and unique production bases of high-quality rice, Jianli County, Qianjiang City, Yijiang City, and Jingshan City in Jianghan Plain were selected as the places for the field survey. Specifically, Jianli County and Qianjiang City are located at the southern part of Jianghan Plain. Superior irrigation and drainage conditions are conducive to develop modern agriculture, and they both are the national modern agricultural reform pilots. Qianjiang City is known as the hometown of shrimp in China, while Jianli County successfully transformed from “China’s first county for rice production” to “China’s first county for shrimp”. In 2017, the area of “shrimp and rice farming” Jianli County and Qianjiang City was 3.33 × 10^4^ hm^2^ [23] and 3.30 × 10^4^ hm^2^ [24], respectively, and they are becoming the core area of rice and shrimp breeding in Hubei Province, accounting for 10% of the country’s total production. Yicheng City and Jingshan City are located in the northern part of Jianghan Plain. They have a large rice planting area, superior water conservancy conditions, and good conditions for agricultural development. Agricultural science and technology demonstration farms that used pesticide control techniques and deep tillage are vigorously constructed in Yicheng, with one of the largest parks taking up approximately 80 hm^2^, in which the use of fertilizer decreased by 10% annually during 2017–2019 [25].Jingshan is the birthplace of Chinese farming culture and unique production bases of high-quality rice (named as *Guo Bao Qiao Mi*). Straw returning to field, no-till, and less-tillage are the three main conservation tillage types applied in Jingshan County. The mechanized pulverization of straw returned to the fields in Jingshan reached 1.06 × 10^5^ hm^2^ in 2018 [26].

### 2.2. Data Collection

The data in this study were collected in the form of questionnaire interviews. The content of the questionnaire consisted of three parts: (1) the respondents’ family socio-economic characteristics, including smallholders’ disposable income, education level, government‘s support policy for conservation tillage, and smallholders’ access to information status; (2) the input and output factors on agricultural land, which mainly includes the planting area, seed consumption, pesticide usage, fertilizer usage, and agricultural film usage; (3) smallholders’ adoption of conservation agricultural technology. It consists of the decision of smallholders to adopt conservation agricultural technology, the degree of adoption of conservation agricultural technology, and the reasons for adoption and non-adoption.

The data used in this study come from a rural householders’ sample survey of 4 places (Jianli County, Qianjiang City, Yijiang City, and Jingshan City) in Jianghan Plain during July to December of 2019. In each surveyed place, the stratified sampling method was applied to select samples. More specifically, based on the economic development level of each township and population size, 3 towns were selected in each place. Then, 3 to4 villages in each town were chosen based on the smallholders’ status of conservation tillage adoption. Finally, 15 farmers in each village were selected through random sampling. In all, 775 sample households were collected in this survey, and 704 (90.84%) were valid. Moreover, the DEA model is very sensitive to outliers, which will cause serious distortion of the results. Therefore, the outliers were excluded in this paper, and 695 smallholders were included in the models finally.

## 3. Research Methods and Variables’ Definition

### 3.1. Three-Stage DEA Model

The DEA model is implemented through two different methods, both of which are based on technical efficiency. The concept of technical efficiency is defined as the DMU (the decision-making unit of production) that produces the highest level of output given the existing technology (output-oriented model), or alternatively, uses the least possible amount of inputs to obtain a given output (input-oriented model) [27]. At the same time, the DEA model can find the optimal weights through linear programming without estimating the production function. It is not affected by the dimension of input and output variables and simplifies the calculation and conversion process [28]. However, the traditional DEA model does not consider the external environmental factors and the impact of random errors in the overall efficiency. Therefore, based on the previous research, a new three-stage DEA model was proposed by Fried [15]. It combines DEA and SFA, using the slack variables of the traditional DEA model as the explained variable and the environmental variables as the explanatory variables. The regression results of the SFA model are used to eliminate the environmental factors and statistical noise in the initial input, and finally the true efficiency is recalculated.

#### 3.1.1. The First Stage: The Traditional DEA Model

At this stage, the traditional input-oriented Banker Charnes and Cooper (BBC) model is applied to optimize the production input factors of smallholders to improve production efficiency, and the initial input–output data of 695 DMUs are used. In the BBC model,

(1) $$\min[\theta -\varepsilon (e^{T}s^{-}+e^{T}s^{+})]$$

(2) $$\begin{array}{l} \sum_{j=1}^{n}\lambda_{j}y_{rj}-s^{+}=y_{r0}\\ \sum_{j=1}^{n}\lambda_{j}x_{ij}+s^{+}=\theta x_{j0}\\ \sum_{j=1}^{n}\lambda_{j}=1\\ \lambda_{j}\geq 0;i=1,2,\cdots ,m;j=1,2,\cdots ,n;r=1,2,\cdots ,s;s^{+}\geq 0;s^{-}\geq 0 \end{array}$$

where *θ* demonstrates the comprehensive operational efficiency value of each DMU, $x_{ij}$ and $y_{rj}$ are the i-th input and r-th output of the j-th DMU, and $\lambda_{i}$ implies a j dimensional weight vector of the DMU j, $s^{-},s^{+}$ are the input slack variable values and output slack variable values for smallholders.

Based on the input-oriented CharnesCooper and Rhodes (C^2^R) and BCC model, the comprehensive total efficiency (TE) can be decomposed into two parts: pure technical efficiency (PTE) based on the variable return to scale (VRS) DEA model and scale efficiency (SE) based on the constant returns to scale (CRS) DEA model. The comprehensive technical efficiency based on the technical reference at time t and t_1_ can be expressed by the following mathematical formula:

Total efficiency = Pure technical efficiency × Scale efficiency.(3)

#### 3.1.2. The Second Stage: Stochastic Frontier Analysis

After the traditional DEA model analysis, the input slacks of all DMUs are influenced by the external environment parameters, managerial inefficiency, and statistical noises. However, at the second stage, they can be eliminated by the stochastic frontier analysis approach. Fried [15] decomposes the redundant variables obtained from the first-stage DEA analysis into the external environment, managerial inefficiency, and statistical noises. He also constructs a similar SFA regression function to strip three factors as follows:

(4) $$S_{ij}=f^{i}(Z_{j};\beta^{i})+\mu_{ij}+\nu_{ij},i=1,2,\cdots ,n$$

where $S_{ij}$ is the slack variable values of the i-th input of the j-th farm household; $Z_{i}$ implies the exterior environmental values; $\beta^{i}$ implies the coefficients of the environmental variables; $\mu_{ij}$ represents managerial inefficiency term; $\nu_{ij}$ illustrates the statistical noises.

Setting $\gamma =\delta_{iu}^{2}/(\delta_{iu}^{2}+\delta_{iv}^{2})$, then the value of γ closes to 1 and demonstrates that the impacts of management inefficiency dominate the investment inefficiencies of DMUs, and thus the SFA approach could be utilized for estimation. Therefore, the value of *γ* is used to identify the feasibility and applicability of the SFA approach for regression.

In order to make all the decision-making units in the same external environment, the regression model eliminated the environmental factors and random disturbances in the original input.

(5) $$\overset{\wedge }{x_{ij}}=x_{ij}+[\max\{Z_{j}{\overset{\wedge }{\beta }}^{i}\}-Z_{j}{\overset{\wedge }{\beta }}^{i}]+[\max\{{\overset{\wedge }{\nu }}_{ij}\}-{\overset{\wedge }{\nu }}_{ij}],i=1,2,\cdots ,m;j=1,2,\cdots ,n$$

where $x_{ij}$ is the input before adjustment; ${\overset{\wedge }{x}}_{ij}$ implies the input after adjustment; $\beta^{i}$ are the coefficients of the environmental variables; $\nu_{ij}$ illustrates the statistical noise.

#### 3.1.3. The Third Stage: Adjusted Efficiency

After adjusting the input slacks in the second stage, the investment value ${\overset{\wedge }{x}}_{ij}$ replaces the original input value $x_{ij}$, and then the BCC model is applied again to recalculate Equation (1). Finally, the real efficiency value excludes the influence of external environmental variables and statistical noise, which is more accurate [15].

### 3.2. Variables’ Definition

#### 3.2.1. Definition of Input–Output Variables

According to the agricultural conditions of the Jianghan Plain and a large number of literature references, the input and output variables are selected to measure agricultural productivity growth by DEA models, as shown in Table 1.six variables for DEA models are used as inputs: planting area, seed consumption, labor input, pesticide usage, chemical fertilizer usage, and agricultural film usage; while agricultural output is regarded as output. The input and output data of the 695 smallholders from the four major cities in Jianghan Plain were collected between July and December in 2019.

The choosing of input and output variables is a critical procedure in the evaluation of DEA efficiency. It requires that all variables grow in the same direction, namely an increase in inputs should lead to an increase in outputs responsively. In order to ensure that the input and output items of the DEA model conform to the “isotropic” assumption, this paper adopts the Pearson correlation coefficient to test the correlation between inputs and outputs. If the correlation coefficients between input items and output items are all positive and pass the 1% explicit significance test, this shows that the selected input–output indicators are reasonable. As shown in Table 1, the selected input and output variables are consistent with the requirements of the DEA model.

#### 3.2.2. Definition of Environmental Variables

According to Zhang et al. [29], agricultural production efficiency is not only affected by the input and output factors but also influenced by the external environmental factor. In addition to the effect of farmland input factors, they also include local socioeconomic development [30], policy environment [5], and information channel [31]. These factors will directly affect the input elements and, thus, affect their agricultural production efficiency. Environmental variables are factors that are not subjectively controlled by the sample farmers in the agricultural production process [29,32,33]. Due to the characteristics that the producers themselves cannot control or change in the short term, these factors are called external environmental factors. According to the research of Wu [34], Xu [35], and Xue et al. [36], this paper selects indexes such as smallholders’ agricultural income, education level of smallholders, policy support, and information acquisition as external environment variables.

Smallholders’ agricultural income often affects their decision-making of production behavior, the degree of information acquisition, and the use of technology. On the other hand, the improvement of economic conditions may increase the degree of concurrent employment of smallholders, which in turn affects agricultural production. The level of education directly determines the scientific and reasonable structure of input factors to achieve the optimal agricultural output of smallholders.Policy support has an important impact on the production behavior of smallholders by ensuring the basic lives of smallholders, improving their production enthusiasm, and increasing income expectations. At the same time, the application of technology and information is inseparable in the process of agricultural modernization. The acquisition of agricultural information resources and the application of advanced technologies have a great role in promoting traditional agricultural production. Based on the above analysis, the descriptive statistical characteristics of the input–output variables and environmental variables for conservation tillage adoption are shown in Table 2.

As shown in Table 2, the interviewee’s agricultural output is 2.55 × 10^4^ kg/hm^2^, and the farming households are large in scale, and the average farming area per household is 1.47 hm^2^. At the same time, the smallholders’ use of seed fertilizers and pesticides during planting is 3500 Yuan/hm^2^. Most growers invest 2 laborers for agricultural production, while most smallholders invest 323.25 Yuan/hm^2^ in agricultural film for agricultural production. Comparing the basic information of the interviewee with the corresponding indicators in the statistical yearbook of the Jianghan Plain, it is found that indicators such as agricultural output, farmland management scale, and labor input are relatively close to the selected sample average.Therefore, the selected samples are highly scientific and representative.

## 4. Empirical Analysis of Smallholders’ Agricultural Production Efficiency

### 4.1. Analysis of Smallholders’ Traditional Agricultural Production Efficiency

In this stage, the DEAP 2.1 software was used to measure smallholders’ agricultural production efficiency without considering the impact of external environment variables. It can be concluded that the total efficiency of agricultural production is 0.465 (0.501 × 0.930), and the pure technical efficiency is 0.501. It is at a low efficiency level (TE, PTE < 0.600). The average scale efficiency is 0.930, which is at a high efficiency level (SE ≥ 0.800). Therefore, the ineffectiveness of pure technology is the main factor that leads to inefficient use of resources. Furthermore, the analysis of the total efficiency shows that the current economic behavior of smallholders in the production process accounts for 53.5% (1–0.456) of resource waste. In other words, under the premise of a basically stable market environment, improving the efficiency of production technology can significantly improve the efficiency of agricultural production andincrease the household income of smallholders. Therefore, in the process of agricultural production in China, the adoption of conservation agricultural technology needs to be maintained at a reasonable ratio of production factors. Because this stage does not consider the impact of external environment variables, random errors, and other factors, it cannot reflect the real investment efficiency of these enterprises. In the second stage, therefore, after eliminating the external environmental variables, random errors, and low management efficiency through the SFA regression model, the original input of smallholders’ agricultural production will be adjusted accordingly.

### 4.2. Analysis of Impact of Environment Variables on Efficiency

At this stage, the SFA model is used to perform regression analysis on the input slack variables (interpreted variables) separated in the first stage including planting area, seed consumption, pesticide usage, chemical fertilizer usage, and agricultural film usage. Four external environmental variables, smallholders’ agricultural income, education level of smallholders, policy support, and information acquisition, are used as explanatory variables. The SFA regression results are shown in Table 3. The log-likelihood function is notable at 1% level in Table 3, which indicates that the regression results are valid. The value of γ of the 6 input variables is close to 1, indicating that the main factor contributing to the inefficiency of DMUs is management inefficiency. Environment variables have strong impacts on the efficiency of smallholders’ productivity. Therefore, to accurately assess the efficiency of smallholders’ productivity, the uncontrollable external environment variables must be eliminated. Since the SFA model uses the input redundancy value as the explained variable at this stage, a positive coefficient indicates that the environmental variable increases the redundancy value, resulting in a decrease in technical efficiency. A negative coefficient indicates that the environmental variable will reduce the redundancy value and improve technical efficiency.

According to Table 3, the impacts of environmental variables on the input variable are listed as follows: (1) The impact of farm households’ agricultural income on planting area, agricultural film usage, and labor input redundancy is significantly positive, indicating that the increase in farm household income will increase the redundancy of input variables and reduce technical efficiency. Due to the irrational behavior of smallholders in the actual production process, the increase in income may cause pure smallholders to blindly expand the planting area in order to increase agricultural income and invest excessive agricultural film in the production process, resulting in waste of resources; relying on part-time work income, smallholders prefer non-agricultural industries with higher returns, which reduces technical efficiency. (2) The educational level of the smallholder has a significantly negative impact on the redundancy of planting area, amount of pesticides, and the amount of agricultural film. This can be explained by the fact that smallholders who have a higher than average education level have the ability to organize more reasonable planting area and design more scientificinputting amounts of pesticides and agricultural film. Therefore, repeated investment can be reduced, and technical efficiency can be improved. (3) The impact of policy support on planting area, seed consumption, labor input fertilizer, and pesticide usage input redundancy is significantly negative. This indicates that although agricultural subsidies have increased the expected income of smallholders to a certain extent, the greater the government’s support for the conservation tillage technology, the better smallholders can reasonably plan the planting area and the input of seed fertilizers and pesticides, thereby reducing redundancy and improving technical efficiency.(4) Information acquisition has a negative impact on the redundancy of chemical fertilizers, pesticides, and agricultural film inputs, and it has passed the 10% significance test, indicating that the more convenient the smallholders’ information acquisition, the more rationally they can plan their agricultural land material expenditures to increase resource utilization. However, smallholders who fail to receive information onconservation agriculture in time have a low technology penetration rate. In actual production, smallholders blindly invest in production factors, leading to a waste of resources.

Since various environmental variables have a significant impact on the redundancy of input elements, it is necessary to adjust the original input to examine the true efficiency level of smallholders.

### 4.3. Smallholders’ True Efficiency

In order to examine whether the efficiency values of the DEA model operation in the first stage and the third stage are consistent, a Wilcoxon rank-sum test was conducted in this study. Suppose H0: the two evaluation results are consistent, H1: the two evaluation results are inconsistent. The result of the above test is shown in Table 4.

From the test results in Table 4, we can see that the sum of negative signs is 152,289.30, the sum of positive signs is 71,156.50, the Z test statistic is −8.130, and the corresponding p value is 0.000. The significance level α is 0.01, which means the p value is less than the significance level α, so the null hypothesis is rejected.Therefore, the efficiency values of each stage are significantly different from each other. This further verifies that the environment and random factors in the original data in this paper have a greater impact on the calculation of the efficiency value, so it is very necessary to create a homogeneous environment by adjusting the amount of input slack.

After excluding the influence of environmental factors and random errors, the adjusted input and original output are brought into the BCC–DEA model, and the DEAP 2.1 software is used to obtain the trueagricultural production efficiency of smallholders, the relevant result of the efficiency values of the first stage and the third stage are shown in Table 5.

Compared with the first stage, after excluding the environment and random factors in the third stage, the total efficiency of the smallholders dropped from 0.465 to 0.434 (0.469 × 0.926). The pure technical efficiency dropped significantly from 0.501 to 0.469, and the scale efficiency dropped from 0.930 to 0.926. It can be concluded that the low total efficiency still comes from the inefficiency of pure technical efficiency. In other words, 56.6% (1–0.434) (as shown in Table 5) loss of the total efficiency comes from the economic behavior of smallholders during the production process. More specifically, the ineffectiveness of pure technology efficiencyof the traditional DEA result in the first stage is more seriously underestimated than that in the third stage. Theoretically, the efficiency values obtained by the DEA model in the third stage can better characterize the smallholders’ trueagricultural production efficiency. Therefore, as a new element of agricultural production, conservation tillage technology can not only change the previous intensive agricultural production model but can also effectively improve the total efficiency of agricultural production. It shows that the scale conditions of the agricultural land of smallholders in the Jianghan Plain are not bad, but the agricultural production technology is relatively low. Based on the above analysis, it is more reliable for the smallholders in Jianghan Plain to improve their agricultural income by adoptingconservation tillage during the agricultural production process.

## 5. Conclusions and Policy Implications

### 5.1. Conclusions

Based on the field survey data of 695 smallholders from the Jianghan Plain, the three-stage DEA model was used to eliminate environmental factors and random factors and to analyze the agricultural production efficiency of smallholders in this study. The specific conclusions of this research are as follows: (1) Using the traditional DEA model to calculate the production efficiency of smallholders in Jianghan Plain, the agricultural production efficiency of smallholders is at a low level in the studied group. In addition, environmental and random factors have impacts on efficiency, so they need to be stripped. (2) External environment variables and random errors have significant impacts on the agricultural production efficiency of smallholders in this study. Compared with the results of the first stage, the drop in pure technical efficiency of smallholders in the third stage is higher than the drop in scale efficiency. It shows that pure technical efficiency is the main factor restricting the agricultural production technical efficiency of smallholders, and the adoption of conservation agricultural technology for smallholders’ agricultural production has not yet reached the optimal level in the studied group. (3) In this study, the educational level of the smallholder, policy support, and information acquisition are conducive toimprove the agricultural production technical efficiency, while farm households’ agricultural income has a negative effect on agricultural production efficiency.

### 5.2. Policy Implications

In order to improve the agricultural production efficiency of smallholders in Jianghan Plain and areas sharing similar edaphic and climatic characteristicsto Jianghan Plain, this article puts forward the following policy implications according to the conclusions in the studied group. (1) At present, the technical efficiency of agricultural production in the Jianghan Plain is still very low, which is mainly due to the low pure technical efficiency of farmers rather than due to scale efficiency. In order to improve agricultural production efficiency, the level of agricultural technology management should be improved. Hence, the government should actively publicize the benefits of conservation tillage to smallholders and carry out scientific agricultural production guiding activities to ensure the smooth implementation of the above technologies. More importantly, appropriate conservation tillage that is adapted to the local agricultural production conditions should be adopted by the smallholders rationally. Only in the above way can the conservation tillage effectively improve the technical efficiency of agricultural production.(2) The education level of smallholders, the government’s policy support for conservation tillage, and the degree of smallholders’ access to conservation tillageinformation are conducive to improving smallholders’ agricultural production efficiency. Therefore, first of all, rural basic education should be strengthened to improve the scientific and cultural quality of smallholders. Second, the agricultural technology service system needs to be promoted—relevant technical training should be actively carried out and the organic combination of smallholders’ planting experience and advanced technology should be promoted. Third, targeted agricultural subsidy policies should be designed to support the smallholders who actively adopt them. (3) Last, but not least, it is necessary to avoid the dissipation phenomenon of continuous advancement of conservation tillage and continuous reduction of technical efficiency. Finally, the adoption of conservation agricultural technology should draw extensively on the experiences of the developed countriesand be combined with the specific conditions of various parts of China.

## Figures and Tables

**Table 1:** Pearson correlation for inputs and outputs.

Variables	Planting Area	Seed Consumption	Labor Input	Pesticide Usage	Chemical Fertilizer Usage	Agricultural Film Usage
**Agricultural output**	0.407 *** (0.000)	0.048 *** (0.000)	0.064 *** (0.000)	0.253 *** (0.000)	0.247 *** (0.000)	0.069 * (0.073)

Note: * indicates 10% significance level; *** indicates 1% significance level; Values in parentheses are p-values.

**Table 2:** Descriptive statistical characteristics of each variable.

Variable Type	Variable Name	Mean	SD
**Output variables**	Agricultural output (kg/hm^2^)	2.55 × 10^4^	820.42
**Input variables**	Planting area (hm^2^)	1.47	1.87
	Seed consumption (Yuan/hm^2^)	5666.7	691.57
	Labor input (person)	2.18	1.57
	Pesticide usage (Yuan/hm^2^)	4468.65	439.27
	Chemical fertilizer usage (Yuan/hm^2^)	5474.1	351.85
	Agricultural film usage (Yuan/hm^2^)	323.25	77.99
**Environmental variables**	Smallholders’ agricultural income (Yuan)	47,380.37	49,100.91
	Education level of smallholders(Year)	2.42	0.90
	Policy support (Yes or No)	1.54	0.50
	Information acquisition (5-Likert Scale)	1.84	0.88

**Table 3:** Results of the stochastic frontier analysis (SFA) regression.

Variables	Planting Area	Seed Consumption	Labor Input	Pesticide Usage	Chemical Fertilizer Usage	Agricultural Film Usage
**Constant term (β0)**	−17.75 ***	−279.94 ***	−274.25 ***	−274.25 ***	−350.42 ***	−34.99 ***
**Smallholders’ agricultural income (β1)**	0.00004 ***	0.00006	0.00003 *	0.00005	0.00002	0.00002 *
**Education level of smallholders (β2)**	−0.02 *	1.49	0.01574	−11.59 *	0.61	−1.93 **
**Policy support (β3)**	−3.69 ***	−63.36 ***	−0.00659 **	−54.10 ***	−63.12 ***	−0.17
**Information acquisition (β4)**	−0.22	1.00	0.00604	−1.75 *	−3.42 *	−0.16 *
**δ2**	−842.76 ***	39,215.74 ***	2.60 ***	20,765.72 ***	19,623.25 ***	8987.48 ***
**γ**	1.00 **	1.00 ***	1.00 ***	1.00 ***	1.00 ***	1.00 ***
**Log-likelihood Function**	−2663.00 ***	−5179.51 **	−857.83 ***	−4612.78 ***	−4593.88 ***	−3507.67 ***
**Likelihood Ratio test**	695.15	649.84	805.81	647.48	635.99	714.02

Note: *, **, *** means passing the significance test of 10%, 5%, and 1%, respectively.

**Table 4:** The results of Wilcoxonrank-sum test

Efficiency Value		N	Rank Mean	Sum of Ranks	Z Test Statistics	Cumulative Probability of Two-Tailed Binomial Distribution
**The first-stage efficiency value**	Negative rank	418 ^a^	364.33	152,289.30	−8.130 ^a^	0.000
**The third-stage efficiency value**	Positive rank	250 ^b^	284.63	71,156.50		
	sum	695				

Note: ^a^ Represents first-stage efficiency value < second-stage efficiency value;^b^ represents first-stage efficiency value > second-stage efficiency value; Z test statistics are statistical results based on negative rank.

**Table 5:** Efficiency values of the first and third stages.

Efficiency Value	Efficiency Value of the First Stage	Efficiency Value of the Third Stage
**The total efficiency**	0.465	0.434
**The pure technical efficiency**	0.501	0.469
**The scale efficiency**	0.930	0.926
